# First identification of porcine parvovirus 6 in North America by viral metagenomic sequencing of serum from pigs infected with porcine reproductive and respiratory syndrome virus

**DOI:** 10.1186/s12985-015-0401-6

**Published:** 2015-10-16

**Authors:** Erin E. Schirtzinger, Andrew W. Suddith, Benjamin M. Hause, Richard A. Hesse

**Affiliations:** Department of Diagnostic Medicine/Pathobiology, Kansas State Veterinary Diagnostic Laboratory, Kansas State University College of Veterinary Medicine, 1800 Denison Avenue, Manhattan, KS 66506 USA

**Keywords:** Porcine parvovirus 6, Porcine reproductive and respiratory syndrome virus, Metagenomic sequencing, North America, PPV6, Pigs

## Abstract

**Background:**

Currently, eight species in four genera of parvovirus have been described that infect swine. These include ungulate protoparvovirus 1 (classical porcine parvovirus, PPV), ungulate tetraparvovirus 2 (PPV3), ungulate tetraparvovirus 3 (which includes PPV2, porcine hokovirus, porcine partetravirus and porcine PARV4), ungulate copiparvovirus 2 (which includes PPV4 and PPV5), ungulate bocaparvovirus 2 (which includes porcine bocavirus 1, 2 and 6), ungulate bocaparvovirus 3 (porcine bocavirus 5), ungulate bocaparvovirus 4 (porcine bocavirus 7) and ungulate bocaparvovirus 5 (porcine bocavirus 3, 4–1 and 4–2). PPV6, the most recently described porcine parvovirus, was first identified in China in late 2014 in aborted pig fetuses. Prevalence of PPV6 in China was found to be similar in finishing age pigs from farms with and without evidence of swine reproductive failure.

**Methods:**

Porcine parvovirus 6 (PPV6) was detected by sequence-independent single primer amplification (SISPA) and confirmed by overlapping and real-time PCR in the serum of porcine reproductive and respiratory virus (PRRSv) positive samples.

**Results:**

Seven nearly complete genomes of PPV6 were identified in PRRSv genotype 2 positive serum samples submitted to state veterinary diagnostic laboratories in 2014. Further testing using overlapping and real-time PCR determined PPV6 to be present in 13.2 % of the serums tested. Additionally, PPV6 was present in samples from all of the geographic locations sampled encompassing nine states in the United States and one state in Mexico. The presence of PPV6 in serum indicates that the PPV6 infection is disseminated and not localized to a specific tissue type. Alignments of the near full length genomes, NS1, and capsid genes identified one of the five PPV6 isolates from China (98.6–99.5 % identity with the North American strains) to be the North American strains nearest relative.

**Conclusions:**

These results are the first to report the presence of PPV6 in North America and demonstrate that the virus is found in multiple geographic areas in the United States and in Mexico. The overall prevalence of PPV6 in PRRSv viremic animals is relatively low. Further, all of the PPV6 genomes found in North America are most closely related to a PPV6 strain first identified in 2014 in healthy pigs from the Tianjin province of China.

**Electronic supplementary material:**

The online version of this article (doi:10.1186/s12985-015-0401-6) contains supplementary material, which is available to authorized users.

## Background

The family *Parvoviridae* consists of many small, non-enveloped, single-stranded DNA viruses that infect a wide variety of species [1]. *Parvoviridae* is further divided into two subfamilies: the *Parvovirinae* that infect vertebrates and the *Densovirinae* that infect invertebrates. *Parvovirinae* is comprised of eight genera; *Copiparvovirus, Tetraparvovirus, Erythroparvovirus, Bocaparvovirus, Dependoparvovirus, Amdoparvovirus, Aveparvovirus* and *Protoparvovirus* [2]. Parvoviruses have a linear genome of four to six kilobases (kb) that contains two major open reading frames (ORFs) encoding the non-structural protein(s) (NSP) and one to four capsid proteins. The protein-coding sequence is flanked by terminal palindromes that fold into duplex hairpin structures that are necessary for DNA replication [1]. The genomic organization of PPV6 is similar to that of other parvoviruses. The nearly full length PPV6 genome consists of negative strand DNA of approximately 6100 nucleotides (nt). There are two putative ORFs separated by eight nt and flanked by 5′ and 3′ UTRs (333–364 nt and 219, respectively). ORF1 encodes a putative non-structural protein (NS1) of 662 amino acids that functions as the viral replicase. ORF2 encodes the putative capsid protein (VP1) that is predicted to be 1189 amino acids in length. Comparison of the predicted lengths of the capsid proteins of porcine parvoviruses shows the capsid protein of PPV6 to be larger than the other swine-associated parvoviruses (data not shown).

Currently, eight species in four genera of parvovirus have been described that infect swine. These include ungulate protoparvovirus 1 (classical porcine parvovirus, PPV), ungulate tetraparvovirus 2 (PPV3), ungulate tetraparvovirus 3 (which includes PPV2, porcine hokovirus, porcine partetravirus and porcine PARV4), ungulate copiparvovirus 2 (which includes PPV4 and PPV5), ungulate bocaparvovirus 2 (which includes porcine bocavirus 1, 2 and 6), ungulate bocaparvovirus 3 (porcine bocavirus 5), ungulate bocaparvovirus 4 (porcine bocavirus 7) and ungulate bocaparvovirus 5 (porcine bocavirus 3, 4–1 and 4.2). Prevalence studies conducted in Europe and the United States have shown that ungulate tetraparvovirus 2, ungulate tetraparvovirus 3 and ungulate copiparvovirus 2 occur in both areas with varying percentages of infected animals depending upon animal age, sample type, clinical status, presence of additional viral agents and time frame. Prevalence of parvoviruses in European pigs ranged from 6.4 % for ungulate tetraparvovirus 3, 9.7 % for ungulate tetraparvovirus 2, 6.4 % for ungulate copiparvovirus 2, and 13.44 % for ungulate bocaparvovirus 2 in samples collected between 2006 and 2011 [3]. In the United States, prevalence of parvoviruses was 14.7 % for ungulate tetraparvovirus 3, 13.6 % for ungulate tetraparvovirus 2, 4.1 and 6.6 % for two virus variants in ungulate copiparvovirus 2 (PPV4 and PPV5), 17.2 – 43.1 % for ungulate bocaparvovirus 2, and 24.2 – 31.9 % for ungulate bocavirus 5 [4–7]. In a retrospective study of dated US samples, ungulate tetraparvovirus 3, ungulate tetraparvovirus 2 and ungulate copiparvovirus 2 positive samples were found dating back to 1998. However, the viral variant of ungulate copiparvovirus 2, PPV5, was only found in samples collected after 2006 [7]. A Chinese retrospective study of ungulate copiparvovirus 2 prevalence in samples collected between 2006 and 2010 found that ungulate copiparvovirus 2 did not circulate in China prior to 2009. After 2009, low levels of ungulate copiparvovirus 2 (2.09 %) were found in samples from clinically healthy and ill animals (0.76 and 2.09 % respectively) [8]. In 2009, the first evidence of porcine bocavirus was found in China during testing of samples from pigs on farms with post-weaning multi-systemic wasting syndrome. 38.7 % of diseased pigs and 7.3 % of pigs without clinical disease were found to have the virus. However, in 2011 a study of asymptomatic pigs from five provinces across China found a prevalence rate of 63.2 and 64.4 % for two viral variants of ungulate bocaparvovirus 2. The same year, ungulate bocaparvovirus 5 was isolated in Northern Ireland. Antibodies to this virus were found in approximately 9 % of 369 pig serum samples tested. Additionally, studies by Opriessnig et al. [5], Blomström et al. [9], and Zhai et al. [10] found that animals showing clinical symptoms of disease (*i.e.* PCV-2, PRRS) were more likely to be positive for ungulate protoparvovirus 1, ungulate tetraparvovirus 2,ungulate tetraparvovirus 3, ungulate copiparvovirus 2, or ungulate bocaparvovirus 2 as well.

PPV6 is the most recent parvovirus of swine to be described [11]. PPV6 was first identified by Chinese researchers in aborted pig fetuses that had tested negative for multiple viruses commonly associated with swine reproductive failure (*i.e.* pseudorabies, PRRSv, PCV2) [11]. Using a PCR approach, pigs of different age groups from farms in four different provinces (Beijing (BJ), Jiangsu (JS), Tianjin (TJ), and Sichuan (SC)) with and without evidence of reproductive failure were screened for the presence of PPV6. PPV6 was found in 50 to 75 % of aborted pig fetuses and piglets (respectively), 15.6 % of finishing pigs, and 3.8 % of sows. Prevalence of PPV6 on farms with and without evidence for reproductive failure was similar at 16.7 % and 13.6–21.7 % [11].

Of the eight described porcine parvoviruses, ungulate protoparvovirus 1 and ungulate bocaparvovirus 5 have been isolated in cell culture. However, only ungulate protoparvovirus 1 has been demonstrated to be a causal agent of porcine reproductive failure [12–14]. In contrast, the newly described porcine parvoviruses have not been isolated in cell culture and their pathogenicity has not been evaluated. However, PPV6 was originally identified as the only pathogen in swine aborted fetuses in China [11]. Many of the newly described parvoviruses are found as co-infections with other well-characterized viruses such as PRRSv (the present study) and PCV2 [5] in pigs showing clinical signs of illness such as post-weaning multisystemic wasting syndrome, PCV2 associated disease or reproductive failure such as fetal death or mummification. The association of ungulate tetraparvovirus 2, ungulate tetraparovirus 3, ungulate copiparvovirus 2 and the recently described PPV6 with clinical disease remains ambiguous due to the detection of porcine parvovirus DNA in both healthy and clinically ill animals. Until these newly described parvoviruses can be cultured or derived from infectious clones the connection between these porcine parvoviruses and disease will remain undetermined.

Here we report the first identification of PPV6 from swine in the United States and Mexico. Seven nearly full-length genomes with high similarity to Chinese PPV6 were found during next generation sequencing of PRRSv positive serum samples. Prevalence of PPV6 within the PRRSv positive dataset was then determined by screening all samples by quantitative PCR (qPCR).

## Results

Serum samples positive for PRRSv by qPCR submitted to the Kansas State Veterinary Diagnostic Laboratory, the Iowa State University Veterinary Diagnostic Laboratory, and the South Dakota State University Animal Disease Research and Diagnostic Laboratory were further analyzed by viral metagenomic sequencing. The Illumina (San Diego, CA) MiSeq data yielded seven samples which produced multiple contigs that showed significant expectation scores (*E* < 1×10^−5^) by BLASTn search to PPV6 sequences described in China in late 2014 [11]. The new genomes have been deposited in GenBank (KR709262-KR709268).

These North American-derived (NA) PPV6 genomes contained two non-overlapping open reading frames (ORF) whose amino acid sequences showed homology to the Parvo non-structural 1 (NS1) superfamily and Parvo capsid by BLASTp search. This search also identified homology between ORF1 of PPV6 and the replicase proteins of other members of *Parvovirinae* and indicated the presence of a AAA+ viral helicase domain in PPV6. The presence of the phospholipase A2 domain (PLA_2_) (amino acids HDXXY) and the calcium binding loop (amino acids YXGXG), features found in many other parvoviruses, was identified in ORF2 by a motif search in CLC Genomics Workbench 7.0 (Qiagen, Valencia, CA). The Chinese PPV6 PLA_2_ domain of amino acids HDIRY was conserved in the NA-derived genomes. This domain is variable in ungulate copiparvovirus 2 viral variants as it is present in PPV5 but absent in PPV4. The calcium loop motif of YXGXR was additionally conserved in the Chinese PPV6 and the NA-derived genomes. This sequence differed from the other shared motifs of YXGXF found in ungulate bocaparvovirus 1, ungulate bocaparvovirus 5, adeno-associated dependoparvovirus A and avian adeno-associated dependoparvovirus 1 and YXGXG of the remaining parvoviruses.

### Phylogenetics

Alignments of the nucleotide sequences of the seven new nearly full-length genomes and 31 complete or nearly full-length genomes encompassing the diversity of genera in the *Parvovirinae*, as well as the amino acid alignments of ORF1 (NS1) and ORF2 (capsid) were evaluated for the best-fit model of sequence evolution in MEGA6 [15]. The best models found under the Bayesian Information Criterion for the genomes was General Time Reversible with gamma distributed rate variation (GTR + G), for ORF1 it was Le and Gascuel with gamma distributed rate variation and proportion of invariant sites (LG + G + I) and for ORF2 it was LG + G + F, where F is the frequency of each amino acid [15, 16]. In all cases, the gamma distribution of rate variation was four categories.

**Fig. 1:**
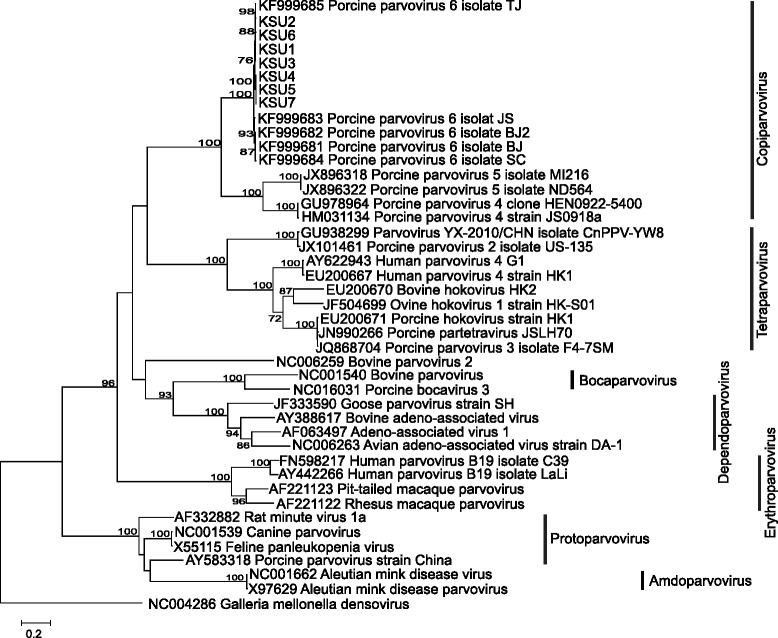
Phylogenetic relationship of seven newly identified parvovirus genomes within *Parvovirinae*. Phylogenetic reconstruction of nucleotide sequences of the newly identified nearly full-length genomes and 31 full-length or nearly full-length genomes downloaded from GenBank that represent the eight genera within *Parvovirinae. Galleria mellonella denosivirus* was used as to root the tree. The tree was reconstructed using the maximum likelihood approach and GTR + G model of sequence evolution as implemented in MEGA6. Nodal support was evaluated by 1000 bootstrap pseudoreplicates. Bootstrap values <70 % are not shown. Scale bars indicate the number of mutations along branches.

**Fig. 2:**
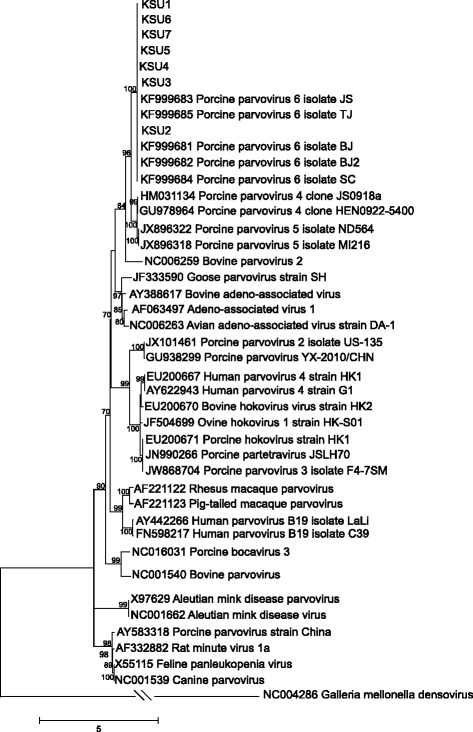
Phylogenetic relationship of ORF1 of seven newly identified parvoviruses within *Parvovirinae*. Phylogenetic reconstruction of amino acid sequences of ORF1 (non-structural protein) using the maximum likelihood approach and the LG + G model of sequence evolution with 1000 bootstrap pseudoreplicates. Bootstrap values <70 % are not shown. Scale bars indicate the number of mutations along branches.

**Fig. 3:**
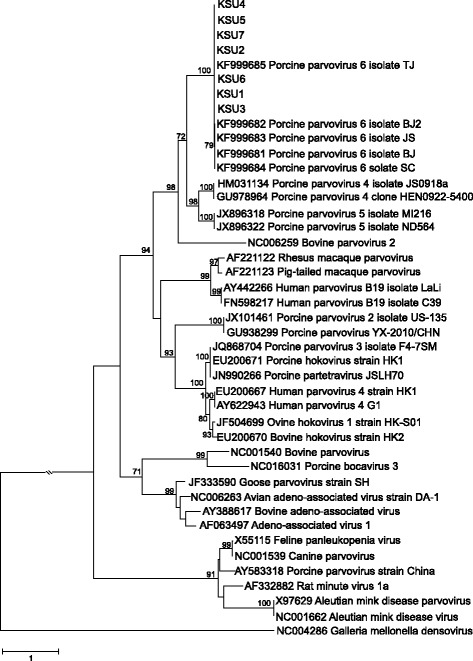
Phylogenetic relationship of ORF2 of seven newly identified parvoviruses within *Parvovirinae.* Phylogenetic reconstruction of amino acid sequences of ORF2 (capsid) using the maximum likelihood approach and the LG + G + F model of sequence evolution with 1000 bootstrap pseudoreplicates. Bootstrap values <70 % are not shown. The scale bars indicate the number of mutations along branches.

The reconstructed phylogenies for each alignment are shown in Figs. 1, 2, and 3. In both nucleotide and amino acid datasets the NA genomes cluster closely with the previously described Chinese PPV6 genomes with very high support. The sister group relationship of the clade consisting of viral variants within ungulate copiparvovirus 2, PPV4 and PPV5, to the clade containing PPV6 is also well supported in all analyses.

### Sequence analysis

Pairwise comparisons of the seven new genomes showed that the NA-derived sequences shared 98.3 – 100 % nucleotide identity with 0 – 106 nucleotide differences. ORF1 and ORF2 showed 99.6 – 100 % (0 – 8 differences) and 99.3 – 99.6 % (15 – 56 differences) nucleotide identity respectively between the NA-derived sequences. Amino acid identities of ORF1 and ORF2 were 99.9 – 100 % (0 – 1 differences) and 99.2 – 99.7 % (4 – 13 differences).

Variation within the Chinese sequences range from 96.8 – 99.9 % at the nucleotide level and 97.4 – 99.9 % in the amino acid sequence of VP1 which is the more variable of the PPV6 ORFs [11]. The NA strains are more closely related to each other (98.9 – 100 % nt and 98.8 – 100 % aa) and the Chinese strain from TJ (98.4 – 99.6 % nt and 98.8 – 99.8 % aa) than the TJ strain is to the other Chinese strains (96.8 – 97 % nt and 97.4 – 97.7 % aa). The greater divergence found in the Chinese strains suggests that PPV6 has evolved over a greater period of time in China as compared to NA. Additionally, the very close homology between the TJ strain and the NA strains suggest that the TJ strain is or is very closely related to the progenitor of the NA strains. Nucleotide identity of the NA genomes with respect to the other four Chinese isolates ranged from 96.9 to 97 % (149 – 193 differences). ORF1 nucleotide comparisons of NA- and Chinese-derived PPV6 genomes showed a 99.1 – 99.9 % identity (2 – 18 differences), while ORF2 comparisons showed 96.1 – 99.8 % identity (7 – 138 differences). Amino acid pairwise comparisons between the NA and Chinese PPV6 ORF1s showed 99.7 – 100 % identity (0 – 2 differences) and ORF2s showed 96.8 – 99.8 % identity (2 – 38 differences).

Comparisons of the genome sequences of the PPV6 clade with its sister clade containing PPV4 and PPV5 found 44.6 – 44.9 % identity with PPV4 and 43.9 – 44.6 % identity with PPV5.

**Fig. 4:**
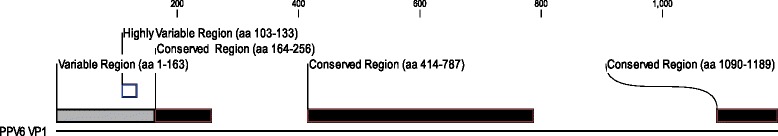
Map detailing the conserved and variable regions of PPV6 VP1. Conserved regions (164–256, 414–787, and 1090,1189) are highlighted in black, the variable region (1–163) is highlighted in grey and the highly variable region (103–133) is highlighted with white outlined in black.

Alignment of the VP1 amino acid sequence identifies only two regions of greater than 100 amino acids in length that are conserved, 415 – 787 and 1090 – C-terminus, there is also a slightly smaller conserved stretch from 164 – 256 (Table 1 and Fig. 4). The conserved region of 164 – 256 comes immediately after a highly variable N-terminal sequence. The N-terminal 163 amino acids of PPV6 have the greatest variability found throughout the entire protein sequence of VP1. There are 18 amino acid substitutions in this region, nine of these substitutions are located within a 31 amino acid stretch between aa 103 and aa 133 (Table 1 and Fig. 4). Of the nine amino acid substitutions in this 31 amino acid region, three substitutions are definitively conserved, two involve proline substitutions, the remaining four substitutions involve substitution of charged amino acids for non-charged amino acids, the most significant being K118L or I. The VP1 sequences, KSU4, KSU5, and KSU7, from the northern plains states (Nebraska and North Dakota) have a Q868T mutation that is not found in PPV6 strains sequenced from any other regions. This mutation may prove useful in the future for tracking the movement of different PPV6 strains. The importance of these amino acids substitutions is currently unknown though three amino acids changes of PPV VP1 are reported to be responsible for the pathogenicity of the Kresse strain of PPV [17]. Whether the amino acid variability in PPV6 VP1 affects the pathogenicity or tissue tropism of the virus has yet to be determined.

**Table 1:** Amino acid substitutions within PPV6 VP1 and sample origin location

	PPV 6 isolate											
	BJ	BJ 2	JS	SC	TJ	KSU1	KSU2	KSU3	KSU4	KSU5	KSU6	KSU7
Origin	BJ	BJ	JS	SC	TJ	AZ	AZ	KS	NE	NE	IA	SD
Amino acid VP1												
5	T	-	S	-	-	-	-	-	-	-	-	-
14	K	-	-	-	R	R	R	R	R	R	R	R
19	T	-	R	-	-	R	-	R	R	R	-	R
26	D	-	Y	-	Y	Y	Y	Y	Y	Y	Y	Y
38	S	-	-	-	F	L	-	L	L	L	-	L
52	N	-	T	-	T	T	T	T	T	T	T	T
58	P	-	-	-	-	-	-	-	-	-	A	-
71	D	E	E	-	-	-	-	-	-	-	-	-
103	G	-	R	-	-	-	-	-	-	-	-	-
107	M	-	-	-	I	-	I	-	-	-	-	-
108	S	-	P	-	P	P	P	P	P	P	P	P
116	K	-	L	-	I	I	I	I	I	I	T	I
117	S	-	Q	-	Q	Q	Q	Q	Q	Q	Q	Q
118	D	-	N	-	N	N	N	N	N	N	N	N
126	Y	-	F	-	F	F	F	F	F	F	F	F
127	D	-	-	-	N	-	-	-	-	-	N	-
133	P	-	-	-	-	T	-	T	T	T	-	T
163	V	-	T	-	-	-	-	T	T	T	-	I
257	A	-	-	-	-	-	-	-	-	-	T	-
289	G	-	R	-	-	-	-	-	-	-	-	-
318	L	I	-	-	-	-	-	-	-	-	-	-
323	T	P	P	P	P	P	P	P	P	P	P	P
324	I	-	L	-	L	L	L	L	I	I	L	I
330	V	-	A	-	A	A	A	A	A	A	A	A
359	T	-	-	-	-	-	-	-	P	P	-	P
360	P	-	-	-	K	K	K	K	K	K	K	K
372	P	-	-	-	-	-	-	-	-	-	-	S
374	P	-	-	-	-	-	-	-	S	S	-	S
382	E	-	-	-	-	-	-	-	K	K	-	K
400	Q	-	-	-	-	-	-	-	H	H	-	H
414	F	-	-	-	-	-	-	-	L	L	-	L
788	S	-	-	-	N	N	N	N	N	N	N	N
789	M	-	-	-	R	R	R	R	R	R	R	R
791	H	-	-	-	S	S	S	S	S	S	S	S
792	H	-	-	-	M	M	M	M	M	M	M	M
815	S	-	-	-	A	A	A	A	A	A	A	A
868	Q	-	-	-	-	-	-	-	T	T	-	T
869	A	-	-	-	V	V	V	-	V	V	V	V
873	G	-	-	-	D	D	D	D	D	D	D	D
919	S	-	-	-	N	N	N	N	N	N	N	N
930	D	-	-	-	-	-	-	-	-	-	E	-
938	K	-	-	-	R	R	R	R	R	R	R	R
941	T	-	-	-	S	S	S	S	S	S	S	S
1013	H	-	-	-	R	R	R	R	R	R	R	R
1038	N	S	-	-	T	T	T	T	T	T	T	T
1042	D	-	-	-	E	E	E	E	E	E	E	E
1089	R	-	-	-	H	H	H	H	H	H	H	H

Origin: *BJ* Beijing, *JS* Jiangsu, *TJ* Tianjin, *SC* Sichuan, *AZ* Arizona, *KS* Kansas, *NE* Nebraska, *IA* Iowa, *SD* South Dakota

Dashes represent amino acid conservation

### qPCR

Following the discovery of PPV6 in some of the PRRSv positive serum samples, all of the samples were analyzed using qPCR to determine the presence of PPV6 in samples at concentrations below the detectable limit of the SISPA sequencing assay and to verify the sequencing results. An additional 15 samples, out of a total of 167 tested, were positive for PPV6 as determined by real-time PCR with CTs ranging from 13.0 – 36.8 (see Table S2). These positive samples were confirmed with overlapping PCR and sequencing of the 441 bp fragment. The prevalence of PPV6 in the serum samples tested was 13.2 % (22/167). PPV6 was detected in PRRSv positive samples from every state tested (Arizona, Illinois, Indiana, Iowa, Kansas, Minnesota, Nebraska, North Carolina, South Dakota, and Vera Cruz, Mexico).

## Discussion

Phylogenetic analysis of seven newly described parvovirus genomes recovered by viral metagenomic sequencing of PRRSv positive porcine serum samples and 31 parvovirus genomes downloaded from GenBank show that these new genomes cluster with high support with the PPV6 genomes described in late 2014 from China and should be considered new strains of PPV6 as indicated by Cotmore et al. [2] within the genus *Copiparvovirus* as discussed by Ni et al. [11].

Although, the samples in this study have no age data associated with them, the prevalence of PPV6 in North America is similar to the prevalence found in finishing pigs in China [11]. PPV6 viremia, as determined by the presence of viral DNA in serum, is similar to the prevalence of PPV1 (14.7 %) and PPV3 (19.2 %) in sera and is more prevalent in North America than PPV4 (5.9 %) and PPV5 (7 %) in sera and in tissues (9.3 %). However, it is drastically lower than that of ungulate tetraparvovirus 3 in sera (72 %) and lung tissue (20.7 %, 40.1 % when PCV2 is also present) [4–7]. While the prevalence of PPV6/PRRSv co-infection is relatively low (13.2 %), the geographic distribution of PPV6 suggests that it is not localized to specific regions of the U.S. as PPV6 was detected in PRRSv positive samples from every state (Arizona, Illinois, Indiana, Iowa, Kansas, Minnesota, Nebraska, North Carolina, South Dakota) and the one region in Mexico (Veracruz) tested.

Despite the noncontiguous geographic dispersion of PPV6 in the United States and Mexico the strains found in North America are very closely related to each other with homology between strains, from different geographical locations, ranging from 98.3 to 100 % at the nucleotide level and 99.2 – 100 % at the amino acid level of ORFs1 and 2. These strains are more closely related to each other than the Chinese strains are to each other at both the nucleotide sequence and amino acid sequence of the predicted proteins, 97.1 – 99.6 % and 97.3 – 99.6 % respectively [11]. The North American strains have the greatest homology with the strain first identified from Tianjin, China having between 98.6 and 99.5 % nucleotide identity with this strain as compared to 96.9 – 97.0 % nucleotide identity to the other Chinese strains.

Among all of the strains identified both in North America and China the sequence diversity is highest in ORF2, the capsid protein. This conforms to the predicted behavior of the virus in response to the host immune system, in that the capsid protein would be under the greatest direct pressure from the host immune system. Since ORF2 mutates at a faster rate than ORF1, mapping the rate of change in ORF2 might provide a method for identifying the timeframe in which PPV6 was introduced into North America.

## Conclusion

These results are the first to report the presence of PPV6 in North America and indicate that the virus is found in multiple states in the United States and found in approximately 13 % of pigs with PRRSv viremia. Further, all of the PPV6 genomes found in North America appear to share a single source virus very similar to the strain first identified in China from healthy pigs in Tianjin in 2014. Additional sampling of PPV6 genomes with good spatial and temporal data in North America and China may elucidate ways in which viruses such as these are transported across the globe and provide insights into improved biosecurity.

## Materials and methods

### Ethics statement

The samples used in this study originated from swine submissions to the Kansas State Veterinary Diagnostic Laboratory, the Iowa State University Veterinary Diagnostic Laboratory, and the South Dakota State University Animal Disease Research and Diagnostic Laboratory. The protocol for this study was approved by the Kansas State University Institutional Biosafety Committee.

### Library preparation and sequencing

Serum samples were centrifuged for five minutes (min) at 7500 rpm to remove any large particulates. One hundred and eighty microliters (μL) of clarified serum was treated with nucleases and incubated at 37 °C for 90 min as described by Allander et al. [18]. Nucleic acid was extracted using the MinElute Viral Spin Kit (Qiagen, Valencia, CA) according to manufacturer’s directions and eluted in 25 μL of nuclease-free water (Life Technologies, Baltimore, MD). Total RNA was converted to cDNA and amplified using sequence-independent-single-primer amplification as described by Allander et al. [18]. Native double-stranded DNA and amplified cDNA was quantified using the Qubit high sensitivity DNA reagent kit (Life Technologies, Baltimore, MD) and diluted to 0.2 nanograms (ng) per μL for library preparation. Libraries were prepared using the Nextera XT DNA Sample Preparation Kit according to manufacturer’s instructions (Illumina, San Diego, CA). Libraries were sequenced using the MiSeq (Illumina, San Diego, CA) and v3 reagents. Paired end reads were demultiplexed and fastq files were created with MiSeq Reporter software (Illumina, San Diego, CA).

Paired end reads for each sample were imported into CLC Genomics Workbench 7.0 software (Qiagen, Valencia, CA). For each sample, reads were assembled into contigs using the De Novo Assembly function with default parameters. Contig consensus sequences were identified by BLASTn against the non-redundant nucleotide database at NCBI. The GenBank accession number with the best E-value was used for reference-based assembly using the entire set of paired reads. Consensus sequences were extracted from the reference-based assemblies and used in phylogenetic analysis and sequence comparisons.

### Phylogenetic analysis

To determine the relationship of the seven PPV6-like genomes within the *Parvovirinae*, thirty-one genomes representing all eight genera were downloaded from GenBank (see Additional file 1: Table S1). A member of the *Densovirinae*, *Galleria mellonella densovirus,* was used as the outgroup. These 31 sequences and the seven new genomes were aligned in ClustalW as implemented in MEGA6 [15] or on the ClustalW2 website (http://simgene.com/ClustalW.) using default parameters. Nucleotide alignments of the genomes, NS1 and capsid and amino acid alignments of NS1 and capsid were evaluated for the best model of evolution in MEGA6 under the Bayesian Information criterion [15]. Phylogenies were reconstructed in MEGA6 for each alignment using Maximum Likelihood and the best-fit model of evolution with 1000 bootstrap pseudoreplicates. After reconstruction, the trees were rooted with *Galleria mellonella densovirus* in MEGA6.

### Sequence analysis

Nucleotide and amino acid sequences of the seven new genomes and the five Chinese PPV6 genomes were aligned in ClustalW and then imported into CLC Genomics Workbench 7.0 (Qiagen, Valencia, CA) for pairwise comparison. Comparisons were made of percent identity and number of nucleotide or amino acid differences. Comparisons were also made with alignments of the new genomes and ungulate copiparvovirus 2.

### PCR

Based on the consensus sequences obtained through viral metagenomic sequencing and the PPV6 sequences in GenBank two pairs of primers were designed, using CLC Main Workbench 7.0 (Qiagen, Valencia, CA). One pair of primers, PPV6-F44/PPV6-R53, amplified a 976 bp segment of ORF 2 and the second primer pair, PPV6-F/PPV6-R, amplified a 618 bp fragment. These two pairs of primers when used together form an overlapping pair of primers which amplify two additional fragments of 618 and 441 bp in length. The reaction mixture was composed of 1 μL of sample, 1 μL of 10 μM of each of the primers, 10.5 μL distilled water, and 12.5 μL GoTaq Green Master Mix (Promega, Madison, WI). The thermal cycling conditions for single product amplification and overlapping amplification were 95 °C for 5 min, 35 cycles of 94 °C 30 s, 51 °C 30s, and 72 °C 1 min, followed by a 7 min elongation step at 72 °C. The presence of amplicons of the appropriate size was determined by electrophoresis and staining with ethidium bromide. A real-time PCR assay was also developed using a pair of detection primers (PPV6 qPCR F/PPV6-R53) and probe designed to bind and amplify a portion of the conserved region of ORF2. The real-time PCR reaction mixture was composed of 1 μL of sample, 0.5 μL of 10 μM probe, 0.5 μL of 10 μM of each primer, 10 μL distilled water, and 12.5 μL of GoTaq Colorless Master Mix (Promega, Madison, WI). The thermal cycling conditions were 95 °C for 5 min, 40 cycles of 95 °C for 15 s, 50 °C for 15 s, and 72 °C for 15 s. Amplification and quantification was performed on a Bio-Rad CFX96 (Bio-Rad, Hercules, CA). Both the overlapping PCR and real-time PCR specificity was confirmed through testing of samples containing ungulate tetraparvovirus 3, ungulate protoparvovirus 1, porcine circovirus type 2 (PCV2), and Torque teno sus virus 1 (TTSuV1). Primer and probe sequences are shown in Table 2.

**Table 2:** Primers and probes used for PPV6 detection

Primer/Probe	Sequence	Position
PPV6-F	ATACGCATCCAATACCCAAT	4925, 4944
PPV6-R	TACTGACATTAGGAGGACCC	5523, 5542
PPV6-F44	GGA GCA GAA AAA CAA ACG A	4390, 4480
PPV6-R53	CCA GAA CAG TAG GCC ATA A	5347, 5365
PPV6 qPCR F	CTC TTC ATA TTC GAACCC	5282, 5299
PPV6 Probe	5′ 6-FAM/CAT CCC CGT /ZEN/ CCC CAT AAC A/3′ IABkFQ	5305, 5323

The NA-derived PPV6 genomes are available in GenBank accession numbers KR709262-KR709268.

## Additional files

Additional file 1: Table S1. List of previously published *Parvovirinae* sequences included in the current study. (XLSX 9 kb) Additional file 2: Table S2. Sequence identifier, State of origin and qPCR Ct for PPV6 and PRRSv infected samples. (DOCX 13 kb)
