# Supplementary material for: First identification of porcine parvovirus 6 in North America by viral metagenomic sequencing of serum from pigs infected with porcine reproductive and respiratory syndrome virus
Source: Virol J. 2015 Oct 16;12:170. doi: 10.1186/s12985-015-0401-6 (PMC4609089; doi:10.1186/s12985-015-0401-6)
Supplement: Additional file 2: Table S2. — Sequence identifier, State of origin and qPCR Ct for PPV6 and PRRSv infected samples. (DOCX 13 kb) [file 12985_2015_401_MOESM2_ESM.docx]

**Table S2 Sequence Identifier, State of Origin and qPCR Ct for PPV6 and PRRSv Infected Samples**

| **Sample ID** | **Location** | **PPV6 CT** | **PRRSv CT** |
| --- | --- | --- | --- |
| KSU1 | Arizona | 19.3 | 20.5 |
| KSU2 | Arizona | 20.4 | 25.0 |
| KSU3 | Kansas | 14.7 | 22.0 |
| KSU4 | Nebraska | 14.6 | 22.9 |
| KSU5 | Nebraska | 17.3 | 20.1 |
| KSU6 | Iowa | 13.0 | 19.6 |
| KSU7 | South Dakota | 24.0 | 25.2 |
| KSU8 | North Carolina | 25.9 | 20.7 |
| KSU9 | North Carolina | 26.0 | 16.6 |
| KSU10 | Minnesota | 28.1 | 20.9 |
| KSU11 | Indiana | 36.8 | 24.5 |
| KSU12 | Illinois | 36.1 | 21.6 |
| KSU13 | Unknown, USA | 33.1 | 18.4 |
| KSU14 | Unknown, USA | 35.3 | 15.9 |
| KSU15 | Vera Cruz, Mexico | 35.5 | 23.0 |
